# Management of fracture-related infection in low-resource settings in Africa: recommendations and guidelines from an international expert group

**DOI:** 10.5194/jbji-11-401-2026

**Published:** 2026-07-09

**Authors:** Loïc Fonkoué, Elizabeth K. Tissingh, Leonard C. Marais, Mbonisi Malaba, Kidanemariam Abrha, Jamie Ferguson, Mario Morgenstern, Olivier Cornu, Maritz Laubscher, Randy Buzisa Mbuku, George W. Galiwango, Matthijs Botman, Justyna Wojno, Mtebe Venance Majigo, Claude Martin Jr., William James Harrison, Alexander Thomas Schade, Kebba Marenah, Malvern Nyamutora, Phiona E. Namale, Vuyisa Mdingi, Martin McNally

**Affiliations:** 1 Department of Orthopedics and Trauma, Yaoundé General Hospital, Yaounde, Cameroon; 2 Department of surgery and specialties, University of Yaounde 1, Yaounde, Cameroon; 3 Experimental and clinical research institute, Université Catholique de Louvain, Brussels, Belgium; 4 Royal National Orthopedic Hospital NHS TRUST, London, UK; 5 King's Global Health Partnerships, School of Life Course and Population Sciences, King's College London, London, UK; 6 Department of Orthopaedics, School of Medicine, University of KwaZulu-Natal, Durban, South Africa; 7 Kijabe Medical Centre, Kijabe, Kenya; 8 Ayder Comprehensive Specialized Hospital, Mekelle University College of Health Science, Mekelle, Ethiopia; 9 Bone Infection Unit, Nuffield Orthopaedic Centre, Oxford University Hospitals, Oxford, UK; 10 Center for Musculoskeletal Infections (ZMSI), University Hospital Basel, Basel, Switzerland; 11 Deformity Correction and Bone Healing Unit (DBU-Basel), Department of Orthopedic and Trauma Surgery, University Hospital Basel, Basel, Switzerland; 12 Orthopaedic and Trauma Department, University Hospital Saint-Luc UCL 1200 – Brussels, Belgium; 13 Neuromuskuloskeletal Laboratory (NMSK), Clinical and experimental Research Institute (IREC), UCLouvain, 1200 Brussels, Belgium; 14 Orthopaedic Research Unit (ORU), Groote Schuur Hospital, University of Cape Town, South Africa; 15 Université Catholique de Louvain, Louvain Drug Research Institute, Pharmacologie cellulaire et moléculaire, Brussels, Belgium; 16 CORSU Rehabilitation Hospital, Kisubi, Uganda; 17 Amsterdam University Medical Centre, Amsterdam, the Netherlands; 18 Global Surgery Amsterdam, University of Amsterdam, Amsterdam, the Netherlands; 19 Lancet laboratories, Microbiology Department, Cape Town, South Africa; 20 Muhimbili University of Health and Allied Sciences, Dar es Salaam, Tanzania; 21 AO Alliance Foundation Chur, Chur, Switzerland; 22 Countess of Chester NHS Foundation Trust, Chester, United Kingdom; 23 Malawi-Liverpool-Wellcome Trust, Blantyre, Malawi; 24 University Hospitals Coventry and Warwickshire, Coventry, United Kingdom; 25 School of Medicine and Allied Health Sciences, University of the Gambia, Banjul, Gambia; 26 Parirenyatwa Hospital, Harare, Zimbabwe; 27 Division of Infectious Diseases and HIV Medicine, Department of Medicine, University of Cape Town, Cape Town, South Africa

## Abstract

**Background:** Fracture-related infection (FRI) represents one of the most challenging complications in trauma care and disproportionately affects patients in low-resource settings, where diagnostic capacity, surgical infrastructure, and access to microbiology and reconstructive expertise are limited. **Methods:** An expert group was convened through the African Bone and Joint Infection Network (ABJIN) under the auspices of the European Bone and Joint Infection Society (EBJIS) and with support from the AO Alliance. Recommendations were developed through a three-step process: (1) a two-part survey assessing current practice and priority needs among clinicians from African countries, (2) an in-person multidisciplinary consensus meeting during the COSECSA Congress (Harare, 2024), and (3) an iterative collaborative review by a wider panel of clinicians from 14 countries. **Results:** The group produced context-adapted recommendations covering prevention, terminology, diagnosis, investigations, holistic patient optimization, antimicrobial therapy, local antibiotic delivery, and surgical management of FRI in low-resource settings. Key themes include the following: management of open fractures; diagnostic pathways prioritizing clinical criteria and intra-operative sampling; antimicrobial strategies reflecting local microbiology and drug availability; affordable local antibiotic carriers; and the importance of soft-tissue management, multidisciplinary collaboration, and centralization of complex cases. Barriers specific to low-resource settings – delayed presentation, restricted microbiology services, limited implant availability, and high burdens of comorbidities – were integrated into the recommendations. **Conclusion:** These consensus-based, context-specific recommendations provide practical guidance for FRI in low-resource settings. They aim to support clinicians in delivering safe, cost-effective care, accepting structural limitations while promoting standardization and audit. Further clinical research from low- and middle-income countries is needed to strengthen the evidence base and refine these recommendations.

## 1 Introduction

Fracture-related infection (FRI) can be devastating for individuals and has a significant socio-economic impact at a population level. It is estimated that there are 1.8 million fracture-related infections worldwide each year (Metsemakers et al., 2024), with the burden being particularly pronounced in low-resource settings. Work from the FRI consensus group has guided practice (McNally et al., 2020; Marais et al., 2024b; Depypere et al., 2020; Metsemakers et al., 2020), and implementation of the recommendations in high-resource settings has standardized care and improved outcomes. However, the evidence base underpinning these recommendations originates from high-income countries with limited representation from other health systems. While the principles are sound, their direct applicability and relevance to low-resource settings are not always clear (Tissingh et al., 2022).

Significant differences between settings – including limited access, implant shortages, limited availability of local antibiotic carriers, lack of advanced microbiology and imaging support, deficient soft-tissue-reconstruction capacity, high prevalence of context specific comorbidities, different microbial and resistance patterns, different clinical features of FRI, and health financing mechanisms – may hamper unmodified adoption of valuable guidelines.

There is growing evidence that advocates for the development of context-appropriate recommendations in the management of FRI – both by incorporating the limited available local evidence and by leveraging the experience of clinicians and researchers working in those environments (Tissingh et al., 2022). There is inherent tension between what are considered to be feasible, optional, and aspirational interventions, and this paper aims to present context-appropriate recommendations for the management of FRI in low-resource settings and in Africa in particular.

## 2 Methods

A group of experts from the African Bone and Joint Infection Network (ABJIN) was brought together under the auspices of the European Bone and Joint Infection Society (EBJIS) and with the support of the AO Alliance. The group is multidisciplinary, with specific expertise in FRI management and experience relating to FRI in Africa. Recommendation development took place in three phases: (1) a survey of current practice and priorities, (2) an expert group meeting, and (3) collaborative document review.

### 2.1 Survey

A survey was developed to gather information to supplement existing literature and information about FRI in Africa. Two 34-item questionnaires were sent out to the ABJIN membership in November 2024 using a Microsoft Forms online link. The first questionnaire focused on existing practice, and the second focused on which recommendations should be included in FRI guidelines. Responses from 86 clinicians in 22 countries were analysed (Tissingh et al., 2025), and a summary was presented at the expert group meeting to help inform the discussion.

### 2.2 Expert group meeting

Exploratory meetings in 2023 and 2024 led to the formation of an expert group, with representatives from LMICs and Europe. The group produced a draft proposal which was circulated to a wider group of interested clinicians for discussion. A 1 d consensus meeting took place during the College of Surgeons of East Central and Southern Africa (COSECSA) annual meeting in Harare, Zimbabwe, in December 2024. A draft document was agreed upon at the end of this meeting.

### 2.3 Collaborative document review

From December 2024 to February 2025, the wider expert group continued collaborative review of the recommendations. The group included orthopaedic surgeons, plastic surgeons, microbiologists, and infectious-disease experts and represented 14 countries including Cameroon, the Democratic Republic of Congo, Ethiopia, Malawi, Netherlands, Kenya, South Africa, Switzerland, Tanzania, Tunisia, Belgium, Uganda, the United Kingdom, and Zimbabwe. All members had direct experience in managing FRI in low-resource settings. Consensus was achieved through iterative discussion and revision during the in-person meeting and subsequent online review. Where disagreement arose, recommendations were revised until broad agreement was reached; formal voting or grading of evidence was not undertaken.

### 2.4 ACTIONS guidelines

In order to facilitate dissemination of the guidelines, standardize approaches, and encourage audit of practice, the recommendations were formulated into an African Consensus for Trauma in Orthopaedics: National Standards (ACTIONS) guideline (AOA, 2025b). The Clinical Guidelines for Fracture-Related Infections became the seventh ACTIONS guideline (AOA, 2025a), available in English, French, and Portuguese.

## 3 Recommendations

### 3.1 Recommendations: prevention

Improved management of open fractures is essential to reduce FRIs. The incidence of FRI following an open fracture is high in all settings, up to 52 % in some case series (Natoli et al., 2025; Schade et al., 2020). There are elements of open-fracture management that may be particularly important, such as early treatment in an appropriate centre (Kock et al., 2025) and engagement with community leaders and traditional bone setters (TBSs) may need to be prioritized to reduce delays.

Surgery should prioritize soft-tissue-friendly approaches, which decrease infection risk, as well as implementation of WHO recommendations such as the use of the surgical safety checklists, which has been shown to significantly reduce surgical-site infection (SSI) rates (Haynes et al., 2009).

### 3.2 Recommendations: terminology

FRI is an infection arising at any time following any fracture. Following the publication of the consensus papers (Metsemakers et al., 2018), the term FRI supersedes terms such as post-traumatic osteomyelitis/osteitis, fracture-fixation-associated infection (FFAI), and SSI. An SSI following a fracture is considered to be an FRI as it is impossible to differentiate between deep and superficial infections after fractures. FRI should be used as the diagnostic term to bring clarity to the clinical entity, facilitate use of appropriate treatment principles, and allow for robust research. Fractures occurring through sites of chronic haematogenous osteomyelitis are relatively common, and their management follows similar principles to those applied in FRI.

### 3.3 Recommendations: diagnosis

FRI should be suspected in all fracture cases, at any time point, where there is delayed bone healing following open fractures, surgically treated closed fractures, or fractures initially treated by TBSs. Patients and carers should be informed about the symptoms and signs of FRI to facilitate early review. A documented history and clinical examination should focus on the confirmatory and suggestive criteria of FRI (Govaert et al., 2020) to make the diagnosis (listed in Table 1). With late presentations and under-treatment of early FRI, clinical confirmatory criteria of sinus tracts and purulence may be particularly relevant in low-resource settings (Fonkoue et al., 2024). Any wound breakdown or dehiscence should be considered to be an FRI. In the absence of clinical confirmatory criteria, in any case of non-union or delayed union, or in the presence of suggestive criteria, FRI should be suspected and investigated accordingly.

**Table 1:** Clinical symptoms and signs of fracture-related infection.

Symptoms (patient reported)	Signs (physical examination)
History consistent with FRI	Fistula or sinus tract
Pain	Purulent drainage or pus
Generalized malaise	Pyrexia
Loss of appetite	Erythema
Malodour	Swelling

Blood tests, microbiological analysis, histopathology, and imaging investigations aid in the diagnosis of FRI and support management. Sinus tract samples or wound swabs for microbiological culture are not recommended as they are likely to grow skin commensals that may not reflect the causative pathogen. Intra-operative sampling is the microbiological diagnostic test of choice.

Table 2 summarizes key investigations. Blood tests help assess the general health of the patient and highlight areas that can be optimized. Serum inflammatory markers have limited use in the diagnosis of FRI (Trenkwalder et al., 2024).

**Table 2:** Summary of diagnostic work-up for fracture-related infection.

Investigation type	Investigation	Purpose
Blood tests	Full blood count: haemoglobin	Identify anaemia to allow pre-op optimization; exclude alternative diagnosis (e.g. malignancy)
	Full blood count: white cell count	Elevated count included in suggestive criteria
	Blood film	Identify sickle cell disease to allow pre-op optimization
	Renal function	Pre-op optimization; baseline value prior to antibiotic start
	Liver function	Pre-op optimization; baseline value prior to antibiotic start
	Viral screen (hepatitis B, hepatitis C, HIV)	Pre-op optimization
	If HIV positive: CD4, viral load	Pre-op optimization
	CRP	Elevated level included in suggestive criteria; monitor trends in septic patients
Imaging	Plain radiographs	Diagnosis; particularly serial x-rays; operative planning
	CT scan	Assess union; identify sequestra; fracture fixation planning
	MRI scan	Diagnosis; assess sinus tract and soft tissues
	SPECT CT	Diagnosis; assess union and sequestra
	PET CT	Diagnosis; assess union and sequestra

Routine X-rays are advocated for in all cases of suspected infection and should be prioritized over more complex and costly imaging modalities (Govaert et al., 2020). Serial radiographs should be used to assess the following: fracture union (or potential for union), signs associated with possible infection such as the presence of necrotic or ischemic bone, loosening of implants, or subtle lucent lines around bone fragments or implants. Where available, CT, MRI, and nuclear imaging may be helpful in diagnosis and to guide surgical management, but they are not essential (Govaert et al., 2020). CT scans are useful to assess fracture configuration, bone healing, and the presence of sequestra to plan debridement. MRI is helpful to delineate the pattern of any sinus tracts and extent of soft tissue involvement. ^18^FDG-PET-CT has high sensitivity for diagnosing FRI, particularly in chronic and complex cases without the presence of confirmatory criteria, and may be helpful in differentiating between localized and segmental bone involvement (Lemans et al., 2019).

### 3.4 Recommendations: classification

**Figure 1:**
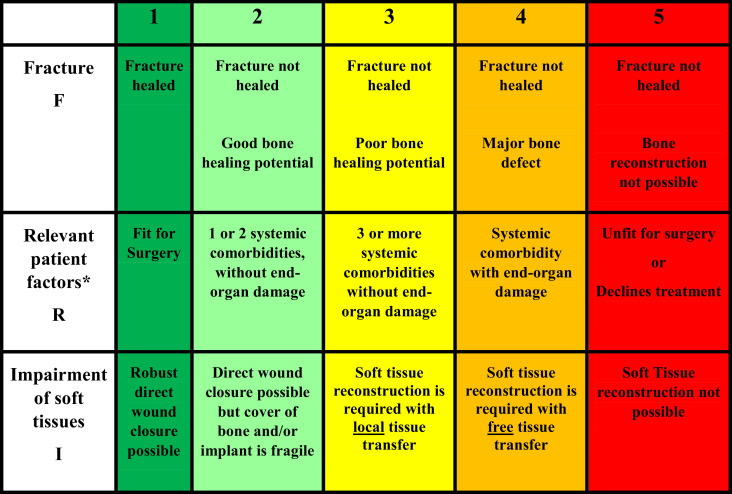
Fracture-related infection classification. Used with permission from © Alt et al. (2024).

The Cierny and Mader classification (Cierny and Mader, 1984) and BACH classification (Hotchen et al., 2019) for osteomyelitis have historically been helpful in guiding the management of bone infection and are validated with regard to outcome. The new FRI classification (Alt et al., 2024) (see Fig. 1) is yet to be fully implemented.

### 3.5 Recommendations: holistic management of established FRI

#### 3.5.1 General treatment principles

There should be a low threshold for instituting prompt appropriate treatment in any case of suspected FRI (Metsemakers et al., 2020). Patients with signs of systemic sepsis require immediate assessment and urgent treatment. For suspected FRI that presents early, prompt intervention is important to ensure good outcomes (Marais et al., 2024a). The treatment goals are fracture consolidation, eradication of infection (or suppression of infection until fracture consolidation is achieved), healing of the soft tissue envelope, restoration of function, and prevention of residual chronic infection.

#### 3.5.2 Medical optimization and patient information

Factors such as low vitamin D, anaemia, thyroid dysfunction, diabetes mellitus, malnutrition, liver function, and renal function should be addressed. HIV management should be optimized (Nieuwoudt et al., 2020). Smoking cessation, alcohol intake reduction and stopping illicit drug use or uncontrolled traditional treatment should be encouraged but should not necessarily preclude treatment. Patients should be involved in the decision-making process, and patients, their family members, and caregivers benefit from being well informed. FRI has a significant associated psychological burden, and patients benefit from a supportive treatment environment and expert input when necessary.

#### 3.5.3 Multidisciplinary teams and specialist units

FRIs are complex and require input from different areas of expertise; often best delivered in the setting of an multidisciplinary team (MDT). The composition of the MDT should be adapted to local resources and expertise; many specialities are available in larger urban centres, and an MDT should, ideally, be set up in facilities managing FRI, as is currently the case for cancers. Studies in high-resource settings have demonstrated better outcomes with MDTs and centralization of services (Ferguson et al., 2021), but further work is needed to demonstrate how this might be applied in low-resource settings.

#### 3.5.4 Antibiotics

Antibiotic treatment should not be started before a diagnostic workup (unless the patient is systemically ill) and should be guided by local prevalence patterns and individual risk factors. Generic broad-spectrum cover should be provided in the peri-operative period and should be rationalized when microscopy, culture, and sensitivity (MC&S) results from intra-operative samples are available. Immediately after surgical sampling, generic intravenous broad-spectrum antibiotics should be given based on local microbial susceptibilities following an agreed-upon protocol and should be continued until definitive culture results are available.

In high-resource settings, *Staphylococcus aureus* has been identified as the most common isolate (Dudareva et al., 2019a; Patel et al., 2023), with no difference in terms of early, delayed, or late FRI (Corrigan et al., 2022). Data from low-resource settings suggest that the microbiology profile may be different, and studies on open fractures and osteomyelitis highlight concerns about resistant organisms and the importance of ensuring that there is Gram-negative cover. Studies on FRI in Cameroon (Fonkoue et al., 2024) and Nigeria (Shodipo et al., 2025) highlight high rates of Gram-negative bacteria: 65.8 % and 49 %, respectively.

For empirical antibiotic treatment (EAT), the association of glycopeptide and an agent against Gram-negative bacteria is recommended (for example: vancomycin and amikacin or meropenem; if these are not available, cefazolin plus gentamicin may be a reasonable alternative) (Fonkoue et al., 2024). Definitive antimicrobial therapy should be culture specific. Drug choice should ideally be made with microbiology and/or infectious-disease expertise and follow principles of good antimicrobial stewardship. In culture-negative FRI, an agreed-upon policy should define the empiric antimicrobial choice.

The choice of antibiotic agent is dependent on sensitivity patterns, bone penetration, oral bioavailability, side effect profile, and biofilm activity. Bacteriocidal agents, with high oral bioavailability and good bone penetration, are generally preferred: rifampicin, fluoroquinolones, clindamycin, co-trimoxazole, tetracyclines (doxycycline or minocyclin), lincomycin, and fusidic acid. Cephalosporins are not recommended as they have lower oral bioavailability. In cases of implant retention, antibiotics with sufficient anti-biofilm activity are recommended. The association of rifampicin (anti-Staphylococcal biofilm activity) + fluoroquinolones (activity on Gram-negative bacteria biofilms) is the treatment of choice, but rifampicin availability is often restricted to TB treatment in many African countries. The combination of rifampicin and clindamycin is generally discouraged as rifampicin induces clindamycin metabolism and may drive inducible resistance. Fosfomycin, which is active against both Gram-negative and Gram-positive bacteria with good bone penetration, is a valuable option as it is available in most African settings, maintains a high sensitivity profile, and can be used in isolation for Gram negatives.

#### 3.5.5 Systemic antibiotic delivery

Oral antibiotic therapy was shown to be non-inferior in relation to intravenous antibiotic therapy (Li et al., 2019). The applicability of the OVIVA to low-resource settings where drug availability, patient expectations, and cost implications are different has not yet been demonstrated.

Antibiotic duration is controversial. Traditionally, 6 weeks of systemic therapy is employed after implant removal and up to 12 weeks when implants are retained. However, recent evidence suggests that shorter and predominantly oral regimens may be sufficient when debridement is adequate and when local antibiotic carriers are used.

#### 3.5.6 Local antibiotic delivery

Antibiotic carriers help deliver antibiotics to the site of the infection, providing targeted delivery and avoiding side effects of systemic administration. In a study of 433 confirmed FRIs, the use of local antibiotics in the definitive management of FRI reduced the rate of failure from 18.3 % to 10.3 % (McNally et al., 2022).

Polymethylmethacrylate (PMMA) is readily available and affordable in African settings. It can be used in spacer or bead form or for implant coating. Disadvantages include the need for multiple-stage surgery to remove the PMMA (which may itself become a foreign body and drive infection) and a poorer elution kinetics profile compared to absorbable carriers, which may lead to its colonization with drug-resistant bacteria. It is usually safe to add the equivalent of a normal daily intravenous or oral dose to a local carrier.

Examples of biodegradable absorbable carriers include calcium sulfate (e.g. Stimulan^©^) and calcium sulfate with hydroxyapatite ceramics (e.g. Cerament^©^, PerOssal^©^). Composite ceramics offer the advantage of being osteoconductive and have an improved drug elution profile. It may be that local aminoglycoside monotherapy is sufficient due to the high local antimicrobial concentrations achieved (Unsworth et al., 2024). Other local antibiotic carriers include intraoperatively made antibiotic-impregnated bone autograft or locally made carriers with calcium sulfate. The use of local antibiotic powder without a carrier is not recommended in the management of FRI as the antibiotic is likely to quickly dissipate into the interstitial fluid, negating its action in established infection (Saka et al., 2024).

Results presented from the SOLARIO trial (Dudareva et al., 2019b) suggest that up to 7 d of systemic antimicrobial therapy is non-inferior in relation to prolonged courses when local antibiotic carriers have been used. This will have significant benefits in compliance with antibiotic therapy and will play a role in improved antimicrobial stewardship. There are, however, no reliable supply chains for these products in most low-resource settings.

### 3.6 Surgical management

The primary objective of surgical management is to restore function, with a healed fracture and eradication of infection. Key surgical steps are illustrated in Table 3.

**Table 3:** Key surgical steps in fracture-related infection management.

Surgical steps in sequential order	Purpose
Sampling	For analysis in microbiology and histopathology laboratories; diagnosis: is it infection? Is there an alternative diagnosis such as malignancy? Microbiology profile to guide antimicrobial options
Debridement	Remove dead and devitalized bone and soft tissues
Lavage	Wash away debris and reduce bioburden
Fracture assessment and stabilization	Support bone union
Insertion antibiotic carrier	Dead space management; antibiotic delivery
Good soft tissue cover	Cover defects and ensure no further environmental contamination; improve blood flow to improve antibiotic delivery and support bone healing

#### 3.6.1 Sampling

Intra-operative sampling is the investigation of choice in FRI, for diagnosis in the absence of clinical confirmatory criteria, and to guide management. For stable patients, antibiotic therapy should be stopped at least 2 weeks prior to sampling (Hoffmann et al., 2024), but a longer period without antibiotics is preferable to improve diagnostic yield (Li et al., 2014). Diagnostic sampling should be carried out as early as possible during surgery, and separate, clean instruments should be used for each sample to avoid contamination. The samples should be sent to an accredited laboratory as soon as possible to improve diagnostic yield. Samples should be sent for microbiology analysis (Dudareva et al., 2021) and histology analysis (Morgenstern et al., 2018). Five samples is the gold standard as the diagnostic yield rises from 84 % for three samples to 97 % for five samples (Dudareva et al., 2021). Where there are resource constraints, three samples is acceptable.

Two or more histology samples of bone and soft tissue should be taken and analysed in an accredited laboratory. In resource-constrained settings, this recommendation could be limited to cases of suspected FRI without clinical confirmatory criteria, and a single histology specimen may be acceptable. The presence of more than five neutrophils per high-power field (NPs/HPF) is significant, and it is a modality that is readily available in low-resource settings, particularly where laboratories carry out blood films for malaria testing.

#### 3.6.2 Excision of dead tissue and irrigation

All non-viable tissue and sequestra should be excised in a meticulous fashion. Viable bone should be preserved to avoid creating a critical bone defect. Bone should be assessed for bleeding, and excision should be progressive until punctuate bleeding (Paprika sign). It may be necessary to create a small elliptical window, with rounded edges to reduce fracture risk, in order to access the intramedullary space. Intramedullary reaming is recommended for extensive medullary infections and the management of infected intramedullary nails.

Copious irrigation with normal saline at low pressure is recommended to decrease the bacterial load and remove debris. The volume of fluid is less important than ensuring all loose fragments are flushed away. Irrigation is deemed to be complete only when the irrigation is flushing through clear with no remaining loose fragments seen in the fluid. The use of additives is not advised as they may add to cell toxicity, and the evidence base is weak regarding their efficacy.

#### 3.6.3 Fracture assessment and stabilization

Following sampling and excision, the bone and fracture should be assessed for healing and stability. This may be done under direct vision of the fracture or using fluoroscopy. If there is any movement detected, the bone should be stabilized as fracture stability is of upmost importance to achieve union and infection eradication.

In FRIs occurring in the first 2–3 months, where there is adequate stability, satisfactory reduction, and good potential for the fracture to heal, prior fixation may be retained. This may be done as part of a debridement and implant retention (DAIR) procedure (Buijs et al., 2022). DAIR may be a particularly useful approach in the low-resource setting where implant availability is restricted.

In late and/or chronic FRI, implants should be exchanged (unhealed fracture) as part of a debridement and implant exchange (DAIEX) procedure or removed (healed fracture). In low-resource settings, when the patient is unable to purchase a new implant, a relevant option is debridement and implant sterilization (DAIS): the implant is removed at the beginning of the surgical procedure, immediately sent for re-sterilization, and reused for fracture fixation after thorough debridement and irrigation during the same procedure.

The exchange of infected intramedullary nails should always be considered due to the difficulty of adequately debriding an infected nail. DAIS may be appropriate. Initial intramedullary nail retention and antimicrobial suppression may be a viable option, particularly if healing is progressing well; however, higher failure rates of infection eradication must be considered. Fracture stabilization with fine-wire circular frames is a good option, but its availability remains limited in many contexts.

#### 3.6.4 Dead-space management and local antibiotic delivery

Bone resection may create a bone defect which is poorly perfused. Approaches to filling bone defects include bone cement with or without antibiotics as part of the induced membrane Masquelet's technique, biodegradable ceramics delivering local antibiotics, antibiotic-impregnated bone grafts (AIBGs), bone allografts, bone transport (distraction osteogenesis) using an external fixator (including Ilizarov frame), and soft tissue transfer such a hemisoleus flap of a vascularized bone graft (fibula free flap).

For stable (contained) bone defects and non-critical unstable conical (uncontained) defects that are healing or expected to heal, normal autologous bone grafting may be a reasonable option in resource-constrained settings. When available, synthetic bone graft substitutes impregnated with antibiotics would also be an option. For critical unstable defects that are not expected to heal and complete (cylindrical) defects, the options are compression alone, compression with distraction at a second site, bone transport, or free fibula grafting. The vascularized fibula flap is often available to be used pedicled in the ipsilateral leg and can be harvested with additional soft tissue such as the flexor hallucis muscle and or a skin island. Other techniques such as the Papineau technique or Lautenbach technique may be reasonable options for healed FRIs in certain settings.

#### 3.6.5 Soft tissue cover

Definitive good-quality soft tissue closure is paramount to improve blood flow, improve antimicrobial delivery, aid fracture healing, and prevent recurrence. A good soft tissue envelope will also help contain local antibiotic carriers. Furthermore, definitive closure of wounds prevents further wound contamination. Local or free flaps may require additional expertise, and it is recommended to include plastic surgical expertise early, as part of surgical planning, if this is available.

There is limited evidence to support specialist dressings, including vacuum-assisted (VAC) dressings, in the management of open fractures and to show that prolonged VAC dressings are detrimental when used to treat FRI (Sweere et al., 2022).

### 3.7 Recommendations: follow-up, evaluation, and outcomes

Regular follow-up is vital to ensure that wounds heal, the fracture unites, and patients continue with their antibiotic therapy (including monitoring for side effects) and to identify early signs of recurrence. Patients should be reviewed by clinicians with FRI management experience for a minimum of 12 months. All patients with proven or suspected FRI should be discussed at regular bone and joint infection MDT meetings wherever possible. There may be particular barriers to follow-up such as geographical distance with poor transport links, cost, and time away from work. Other mechanisms may be considered such as involving community health workers and using messaging systems. Outcomes should be evaluated regularly; measures include re-operation rates, non-union, infection recurrence, amputation, and death.

## 4 Discussion

The recommendations presented in this paper are limited by a lack of strong evidence from low-resource settings in certain key areas, particularly with regard to an MDT approach, local antibiotic delivery, and surgical strategies where implants may be retained. Wherever possible, we have presented the existing evidence base and commented on its validity. There is, however, the potential for bias due to limited evidence.

More research is urgently needed, including on policy implications and to set research priorities. Issues that need urgent consideration include how to build context-appropriate treatment networks and multidisciplinary teams, appropriate antibiotic regimens, optimal sampling recommendations, and how to create supply chains for local antibiotic carriers.

Clinical contexts vary, and, even within low- and middle-income countries, there is significant variation. The principles have been outlined here and should be adapted to individual contexts whilst providing the highest standard of care possible. Robust data collection, regular audit, and reviews of practice should be used to evaluate the implementation and efficacy of guidelines and recommendations.

Whilst FRI treatment should be tailored to the resources available, ministries of health, clinicians, and industry should work together to improve supply chains of vital resources and equipment, including local antibiotics and circular fixators. Resources should also be invested in upskilling clinicians in vital orthopaedic and plastic surgery techniques to create well-staffed specialist units and high-volume centres.

## 5 Conclusion

The burden of FRI is disproportionately borne by patients in low-resource settings. By combining existing evidence with frontline clinical experience, this consensus offers practical, context-specific recommendations designed to improve care despite structural and resource constraints. These guidelines do not replace international standards; instead, they translate essential principles into feasible strategies for hospitals facing shortages of implants, limited microbiology capacity, and high burdens of comorbidity.

Sustainable improvement will depend on investment in training, soft tissue reconstructive capacity, diagnostics, and supply chains, alongside the expansion of multidisciplinary infection teams. Critically, locally generated evidence is needed to validate and further develop these recommendations. Strengthening capacity is essential to ensure that future FRI management guidelines reflect the realities of the settings where they are most needed.

### Key Messages

- FRI is common in low-resource settings and often presents late, requiring clinicians to rely on pragmatic diagnostic pathways prioritizing clinical criteria and intra-operative sampling rather than advanced imaging or laboratory testing.
- Early and appropriate management of open fractures – including timely debridement, antibiotic administration, soft tissue protection, and stabilization in appropriate centres – is the most effective strategy to reduce FRI incidence.
- Antibiotic therapy must be rationalized, guided by local microbiology and resource availability. Adequate debridement with implant removal allows for shorter systemic treatments, while implant retention requires prolonged antibiotic courses and anti-biofilm activity.
- Surgical principles remain central: thorough debridement, stable fixation, appropriate dead-space management, and reliable soft tissue cover. When advanced reconstructive options are unavailable, surgeons should use context-adapted techniques, including local antibiotic delivery and external fixation.
- Multidisciplinary collaboration and system strengthening – including centralization of complex cases, improved supply chains for essential implants and antibiotics, and enhanced training in infection and soft tissue management – are essential to improving outcomes and reducing recurrence.

## Supplement

10.5194/jbji-11-401-2026-supplement The supplement related to this article is available online at https://doi.org/10.5194/jbji-11-401-2026-supplement.

## Data Availability

Primary data referenced in this paper is available in the cited references.
