# Supplementary material for: Management of fracture-related infection in low-resource settings in Africa: recommendations and guidelines from an international expert group
Source: J Bone Jt Infect. 2026 Jul 9;11(4):401–11. doi: 10.5194/jbji-11-401-2026 (PMC13360743; doi:10.5194/jbji-11-401-2026)
Supplement: The supplement related to this article is available online at https://doi.org/10.5194/jbji-11-401-2026-supplement. [file jbji-11-401-2026-supplement.pdf]

Supplement of J. Bone Joint Infect., 11, 401–411, 2026
https://doi.org/10.5194/jbji-11-401-2026-supplement
© Author(s) 2026. CC BY 4.0 License.

Supplement of

Management of fracture-related infection in low-resource settings in Africa: recommendations and guidelines from an international expert group

Loïc Fonkoué et al.

Correspondence to: Elizabeth K. Tissingh (elizabeth.tissingh@kcl.ac.uk)

The copyright of individual parts of the supplement might differ from the article licence.

Management of fracture-related infection in low-resource settings

ACTIONS | African Bone & Joint Infection Network ABJIN | AO ALLIANCE

Background and rationale: Fracture-Related Infection (FRI) is an infection arising at any time following a fracture. It creates a major burden in countries with limited healthcare resources.

Standards of Practice

1. Prevention of FRI

Hospitals should use an agreed-upon and accessible policy for the prevention of infection in open and closed fractures. Open fractures should be managed according to the open fracture guidelines[1].
Patients and carers should be informed about the symptoms and signs of FRI to facilitate early review.

2. General Considerations

FRI is common and should be considered at all time points after fracture.
Patients with signs of systemic sepsis require an immediate assessment and urgent treatment.
For suspected early FRI, early intervention is important to ensure good outcomes.
In late/chronic FRI, patient optimization and treatment should be planned; ideally within a multi-disciplinary team.
Patients should be informed about their condition, treatment plan, follow-up, and expected outcome.

3. Diagnosis and investigation of suspected or confirmed FRI

Antibiotic treatment should not be started before a diagnostic workup (unless the patient is systemically ill)[2].
For stable patients, antibiotic therapy should be stopped at least 2 weeks prior to sampling.
Documented history and examination should focus on the confirmatory and suggestive criteria of FRI[3].
Blood tests (FBC, CRP, U&E) allow assessment of general patient health but do not diagnose or exclude FRI.
Serial radiographs should be used to assess fracture union, implant loosening, periosteal reaction, and progressive bone loss. Sinus tract samples or wound swabs for microbiological culture are not recommended.
Intra-operative sampling is the diagnostic test of choice. Between 3 and 5 microbiology samples and 1 histology sample of bone and soft tissue should be taken using separate instruments and transported immediately to an accredited laboratory.
In patients unable to undergo surgery, for any reason, aspiration of fluid collections or joint effusion for microbiological culture may be appropriate.

4. Management of FRI

A documented pathway of care is essential. The most cost-effective treatments should be given.
Patients with systemic or limb-threatening sepsis must start IV fluid and antibiotics within 2 hours of presentation. Blood cultures should be taken prior to antibiotic administration.
The treatment of FRI usually requires surgery. If this is not possible, suppressive antibiotics may control systemic or local symptoms but rarely eradicate infection. This decision should be documented with an agreed-upon duration.
Systemically well patients should undergo host optimization prior to definitive FRI management.
Early infections can be managed in a unit that provides operative fracture care. The timing of intervention is dependent on the possibility of implant retention. Key steps are:

- Diagnostic sampling as early as possible during surgery, followed by excision of non-viable tissue.
- Fracture stabilisation: all unstable FRIs should be stabilised with external or internal fixation.
  - Stable prior fixation, with satisfactory reduction, may be retained.
  - Always consider exchange of infected intramedullary nails.
  - Inadequate prior fixation should be removed and replaced.
- Definitive good quality soft tissue closure is required (include plastic surgical expertise when available).

Infected non-unions, FRIs with major bone and/or soft tissue defects, or recurrent chronic FRIs are complex. Referral of complex cases should ideally be made to an MDT with access to specialists in ortho-plastic surgery, microbiology, infectious diseases, and radiology.

Immediately after surgical sampling, intravenous broad-spectrum antibiotics should be given based on local microbial susceptibilities in an agreed-upon protocol.

The use of antibiotics delivered locally in bone, in a suitable antibiotic carrier, is advisable. It is usually safe to add the equivalent of a normal intravenous dose to a local carrier, if available.

Definitive antimicrobial therapy should be culture specific. Drug choice should ideally be made with microbiology/ infectious disease expertise, when possible, and follow principles of good antimicrobial stewardship. In culture-negative FRI, an agreed-upon policy should define the empiric antimicrobial choice.

5. Monitoring and follow up

Patients should be reviewed by clinicians who have experience in FRI, for a minimum follow-up period of 12 months.
All patients with proven or suspected FRI (before or after treatment) should be discussed at regular bone and joint infection MDT meetings.
Significant adverse effects from antimicrobial therapy are common and treatment should be monitored.

6. Evaluation and outcomes

Hospitals should have a robust surgical site infection surveillance system. Outcomes should be evaluated regularly.
Primary outcome measures include re-operation rates, non-union, infection recurrence, amputation, and death.

1. AO Alliance, COSECSA, WACS (n.d.). ACTIONS clinical guideline: The Management of Open Tibial Shaft Fractures
2. National Institute for Health and Care Excellence (2024): Suspected Sepsis: Recognition, Diagnosis and Early Management
3. McNally M. et al. (2020). Definition and diagnosis of fracture-related infection. EFORT Open Reviews 5(10), 614-619. DOI: 10.1302/2058-5241.5.190072
